# Neurocognitive function among HIV-infected children on protease inhibitor -based versus non-protease inhibitor based antiretroviral therapy in Uganda: a pilot study

**DOI:** 10.1186/s12887-021-02676-2

**Published:** 2021-04-26

**Authors:** Damalie Nalwanga, Victor Musiime, Paul Bangirana, Erika Phelps Nishiguchi, Andrew Kiggwe, Titus Ssesanga, John M. Ssenkusu, Philippa Musoke, Sarah E. Cusick

**Affiliations:** 1 Department of Paediatrics and Child Health, School of Medicine, College of Health Sciences, Makerere University, P. O. Box 7072, Kampala, Uganda; 2 Makerere University Lung Institute, P. O. Box 7749, Kampala, Uganda; 3 Research Department, Joint Clinical Research Centre, P. O. Box 10005, Kampala, Uganda; 4 Department of Psychiatry, School of Medicine, College of Health Sciences, Makerere University, P. O. Box 7072, Kampala, Uganda; 5 Department of Paediatrics, Division of Developmental Behavioural Paediatrics, University of Washington, Seattle, Washington USA; 6 Department of Epidemiology and Biostatistics, School of Public Health, College of Health Sciences, Makerere University, P. O. Box 7072, Kampala, Uganda; 7 Department of Paediatrics, University of Minnesota, Minneapolis, MN USA

**Keywords:** Neurocognitive function, Children, HIV, ART

## Abstract

**Background:**

HIV infection is associated with significant neurocognitive deficits making maximization of cognitive function among children receiving antiretroviral therapy (ART) a public health imperative. Non-protease inhibitors (non-PIs) achieve higher drug levels in the cerebral spinal fluid (CSF) compared to PIs, potentially leading to better neurocognitive function by reducing CSF viral load and inflammation. ART that maximises children’s neurodevelopment and school achievement could result in improved quality of life and productivity as adults, but little research to date has examined whether non-PI ART is associated with better neurocognitive outcomes. We compared the neurocognitive function between children living with HIV receiving PI-based and non PI-based ART.

**Methods:**

We recruited a consecutive sample of clinically stable Ugandan children living with HIV aged 5–12 years who received PI-based or non PI-based ART for ≥ 1 year (viral load < 1000 copies). Neurocognitive function was assessed using the Kaufman Assessment Battery for Children, the Test of Variables of Attention, and Bruininks-Oseretsky Test of Motor Proficiency. Age-adjusted neurocognitive z-scores for the two groups were compared using linear regression models in STATA version 13. The Hommel’s method was used to adjust for multiple testing.

**Results:**

We enrolled 76 children living with HIV; 34 on PI ART and 42 on non-PI ART. Mean (±SD) age was greater in the non-PI vs. PI group (9.5 ± 1.9 vs. 8.5 ± 2.0) years (*p* = 0.03). Children in the non-PI group had lower socioeconomic scores (5.7 ± 3.3 vs. 7.4 ± 2.8, *p* = 0.02). There was no difference in neurocognitive function between the groups (adjusted *p* > 0.05) for KABC and TOVA. Children in the PI group had better total BOT scores than their counterparts (46.07 ± 1.40) vs. 40.51 (1.24), *p* = 0.03).

**Conclusions:**

We detected no difference in neurocognitive function among children on PI and non PI-based ART therapy based on KABC and TOVA tests. Children on PI based ART had better motor function than their counterparts. We recommend a prospective study with a larger sample size.

**Supplementary Information:**

The online version contains supplementary material available at 10.1186/s12887-021-02676-2.

## Introduction

HIV infection has been associated with significant motor and cognitive deficits among children and adolescents, even in those with high CD4 cell count [1–8]. Lifesaving antiretroviral therapy (ART) has become more widely available to these children and has significantly reduced opportunistic infections, allowing many infected children to thrive. With improved survival of HIV-infected children on ART, maximizing cognitive function to improve their quality of life and economic potential becomes imperative. The central nervous system (CNS) penetration of individual antiretroviral drugs (ARVs) varies. Protease inhibitors (PIs), such as lopinavir, achieve lower drug levels in the cerebrospinal fluid (CSF) compared to reverse transcriptase inhibitors (non PI), such as efavirenz and nevirapine [9]. Persistence of detectable levels of HIV copies in CSF among patients who are serologically suppressed has been described in patients on PIs due to their low CNS penetration when compared to other antiretroviral drugs [10]. Although ART has been documented to improve neurocognitive function of children living with HIV [11–15], the difference in CNS drug penetration could result in a difference in neurocognitive benefit among these children. Bangirana et al found no neurocognitive differences among Ugandan children who had previously been randomized to PI vs. non-PI based therapies [16]. However, this was a cross-sectional study done 5 years after the study participants, who had been initially randomized to receive PI-based ART, had resumed non-PI- based ART, which was the standard of care at the time. It is therefore not clear if the children on PI- based ART had any difference in neurocognitive function over those on non-PI-based ART at the time when they were receiving different ART regimens. ART that maximizes children’s neurodevelopment and school achievement should be established and preferentially chosen for treatment of HIV positive children globally. This could improve the quality of life for children living with HIV, and increase their productivity as adults. This pilot study aimed to compare the neurocognitive function of children living with HIV receiving PI-based to those receiving non-PI based ART and explore the possibility of conducting a prospective study to investigate these differences.

## Materials and methods

### Study setting

The study was conducted at the Joint Clinical Research Centre (JCRC), Kampala, Uganda. It is located approximately 10 km outside Kampala, the capital city of Uganda. JCRC has led the treatment of HIV/AIDS and opportunistic infections in Uganda. It has served over 200,000 clients on first, second and third line ART countrywide. The JCRC paediatric clinic provides longitudinal clinical care and psychosocial support to over 1000 children living with HIV. JCRC also conducts research in several fields including HIV vaccines, ART, opportunistic infections, public health and social behaviours [17]. JCRC is funded through research and implementation grants, institutional research collaborations, internally generated revenue, and support from the Uganda Ministry of Health.

### Study design

We conducted a cross-sectional pilot study among a cohort of Ugandan children living with HIV aged 5 to 12 years receiving protease inhibitor (PI) based ART or non-PI based ART. This age-group was chosen to maximize the number of eligible children on PI-based ART, which was introduced later than non-PI based ART, to enable use of similar neurocognitive tests in the study population.

### Study procedure

We enrolled a convenient sample of 76 virologically suppressed children attending the clinic between March and August 2019 for regular follow up into the study (34 on PI-based ART and 42 on non PI-based ART). Inclusion criteria were: 1) confirmed diagnosis of HIV (based on JCRC clinic records); 2) initiated on ART within the first 5 years of life; 3) received ART for ≥1 year at the time of enrolment; 4) virologically suppressed, defined as viral load less than 1000 copies per ml within the last 6 months prior to screening (based on JCRC clinic records); and 5) aged 5–12 years at the time of screening. Exclusion criteria were: 1) acute illness; 2) current opportunistic infection; 3) temperature > = 38.0 °C at the time of screening; 4) sensory impairment, e.g. hearing or sight, by caregiver report; 5) known cerebral palsy or history of other CNS infection/event; or 6) acute malnutrition (clinically defined as bilateral pitting edema or extreme wasting); 7) caregiver-reported ART treatment adherence less than 80% (calculated as the percentage of prescribed pills consumed); 8) history of receipt of both PI based and non-PI based ART since diagnosis and 9) non-English or Luganda speaking (could not communicate effectively with neurocognitive tester). Of the 93 potentially eligible participants screened, 17 were excluded. Four had either cerebral palsy or history of other CNS infection, 1 had acute illness or opportunistic infection, 5 had received both PI an d non-PI based ART since diagnosis, 2 had sensory impairment, 1 had received ART for less than 1 year, 1 was non-English or Luganda speaking (could not communicate effectively with the neurocognitive tester), and 3 declined participation in the study.

### Data collection

Caregivers of children who attended the JCRC paediatric clinic were informed about the study by the study nurse. Interested caregivers were verbally consented for screening. Written informed consent was obtained from eligible participants’ caretakers, and assent from children 8 years and above prior to enrolment into the study. A pre-tested questionnaire was administered by the study doctor to collect participants’ baseline data including age, sex, years of schooling, anthropometric measurements, prematurity at birth based on estimated gestational age, duration on ART, previous ART, CD4 cell count, and treatment adherence by self-report. Neurocognitive assessments were then carried out for enrolled participants by an experienced neurocognitive tester who was blinded to the participants’ ART regimen. The test scores were crosschecked by another neurocognitive tester for completeness and errors. All neurocognitive assessments were done in the morning because children’s concentration diminishes as the day progresses.

### Neurocognitive test instruments in the study

The Kaufman Assessment Battery for Children, second edition (KABC-II) assesses sequential processing (memory), simultaneous processing (visual-spatial processing and problem solving), learning ability (immediate and delayed memory), and planning ability (executive reasoning). Summation of these four indexes gives the mental processing index which was the primary outcome of the test. The examiner initially scores the child in the different subtests to provide raw scores. The raw scores are then converted to scaled scores ranging from 1 to 19. The sums of the scaled scores create the four indexes mentioned above. From these, standard scores are obtained, and their sum provides the mental processing index (MPI) [18]. The Test of Variables of Attention (TOVA) is a computerised test which measures the child’s impulse control, process inattention, and response time and D prime which was the TOVA’s primary outcome. The Bruininks-Oseretsky Test of Motor Proficiency, second edition (BOT-II) assesses children’s motor skills i.e. balance, coordination, visual-motor control, strength and agility, with the total score of these domains as the primary outcome. The examiner scores the child based on their performance in these tasks. The KABC**-**II was validated by Bangirana et al. among Ugandan children with a history of cerebral malaria [19], and has been extensively used to assess neurocognitive function in HIV infected children in sub-Saharan Africa [8, 13, 20, 21]. The BOT and TOVA have been widely used among Ugandan children with HIV and cerebral malaria [13, 20, 22]. This choice of instruments was based on the effect of HIV on attention, motor function, visual spatial processing, memory and language of children in sub-Saharan Africa [8, 20, 21].

### Sample size

Sample size for this pilot study was based on mental processing index (MPI) of the KABC-II among Ugandan children with HIV. Using the Satterthwaites t test for sample size comparing two independent means with unequal variances [23], with a sample size of 76 children, 42 in the non-PI based ART group and 34 in the PI based ART group, at 5% level of significance, we had 80% power to detect a minimum mean difference of 0.87 in MPI z-scores between children on PI-based and non-PI based ART.

### Data management and statistical analysis

The questionnaires were completed by study staff and reviewed weekly for accuracy and completeness prior to data entry. Data was entered into an electronic database using Epidata version 3.1 software package with built-in quality control checks. The data was double-entered and validated by two entrants. The final data was backed up and exported to Stata version 14.1 (STATA CORP, TEXAS USA) for analysis. Continuous variables were summarized using means and standard deviations for normally distributed data. Categorical variables were summarized using frequencies, and percentages. Linear regression was used to test the association between independent variables (demographic and clinical characteristics) and continuous outcome variables (mean differences).

Participants’ raw scores were compared to scores of healthy children (controls) from a conducted in Uganda in 2008 to 2015 [22] to generate Z scores. The value of the Z-score describes how far participants’ scores deviate from the mean (Z = 0), with positive and negative scores indicating that the score is higher or lower than average respectively. We compared the mean differences in KABC and TOVA performance measures standardised for age between the PI-based and non-PI-based ART groups after adjusting for socioeconomic status, MUAC, WHO stage at initiation of ART, co-trimoxazole prophylaxis and prematurity at birth. Adjusters in the models were chosen based on independent variables for which *P* < 0.20. In addition, the child’s age was excluded from the models since z-scores are already age-adjusted. We also compared raw outcome scores for BOT since we did not have data for healthy community children for standardisation. The scores were adjusted for age, socioeconomic status, MUAC, WHO stage at initiation of ART, co-trimoxazole prophylaxis or prematurity at birth. We considered multiple statistical tests among the sub-scales of a testing tool as one experiment and therefore controlled for the family wise error rate within a testing tool using the Hommel’s method [24]. *P*-values less than 0.05 were then considered statistically significant.

## Results

### Participant clinical and demographic characteristics

Participants in the PI-based ART group (Lopinavir/ritonavir) were significantly younger than those in the non-PI based group (Efavirenz and Nevirapine), 8.5 years (SD ± 2.0) versus 9.5 years (SD ± 1.9), *p* = 0.03. The majority of the study participants in each group were female. More participants in the PI group started ART at age less than 3 years (*n* = 31, 91%) compared to those in the non-PI group (*n* = 26, 62%), *p =* 0.03 (Table 1). Participants in the PI-based ART group scored higher on the socioeconomic status score compared to their counterparts in the non-PI based ART group (7.4 ± 2.8 versus 5.7 ± 3.3, *p* = 0.02). There was no difference in the child years in school, MUAC or BMI for age between the two groups (Table 1). More children in the PI group were WHO stage 1 at initiation of ART than children in the non-PI group, *p* = 0.023, and nearly all children on each group were receiving co-trimoxazole prophylaxis (Table 1).

**Table 1:** Demographic and clinical characteristics of HIV infected children on antiretroviral therapy

Characteristic	ART regimen		***P***-value
	PI based (*n* = 34)	Non-PI based (*n* = 42)	
Age in years, mean ± SD	8.5 ± 2.0	9.5 ± 1.9	0.03
Female sex, No. (%)	20 (58.8)	24 (57.1)	0.88
Child years in school, mean ± SD	5.4 ± 2.5	5.7 ± 1.8	0.52
Socioeconomic status, mean ± SD	7.4 ± 2.8	5.7 ± 3.3	0.02
BMI for age, mean ± SD	−0.6 ± 1.0	− 0.4 ± 1.1	0.45
MUAC, mean ± SD	18.4 ± 1.8	19.0 ± 1.7	0.14
Duration of ART, mean ± SD	7.8 ± 1.7	7.7 ± 1.8	0.84
Primary care giver, No. (%)			0.58
Mother	24 (70.6)	32 (76.2)	
Others	10 (29.4)	10 (23.8)	
Primary care giver education level, No. (%)			0.40^a^
None	–	2 (4.8)	
Primary	16 (47.0)	21 (50.0)	
Secondary	14 (41.2)	11 (26.1)	
Tertiary	4 (11.8)	6 (14.3)	
Don’t know	–	2 (4.8)	
WHO stage at ART initiation, No. (%)			0.02^a^
I	18 (52.9)	9 (21.4)	
II	7 (20.6)	19 (45.2)	
III	8 (23.5)	12 (28.6)	
IV	1 (2.9)	2 (4.8)	
On Co-trimoxazole prophylaxis, Yes. (%)	34 (100)	38 (90.5)	0.12^a^
Child born before term, No. (%)			0.12^a^
Yes	3 (8.8)	–	
No	30 (88.2)	41 (97.6)	
Don’t Know	1 (2.9)	1 (2.4)	
Age at ART initiation			
≤ 3 years	31 (91%)	26 (62%)	
> 3 years	3 (9%)	16 (38%)	0.003

*Abbreviation*: *PI* Protease Inhibitor.

^a^Fisher’s exact test was used to test for associations; otherwise the chi-square test was used. For continuous variables, a t test was used to test for differences in means between groups

### Neurocognitive function among the participants

For the KABC-II, there was no difference mental processing index (*p* = 0.29), sequential (*p* = 0.05) or simultaneous processing (*p* = 0.19), learning (*p* = 0.46), or planning (*p* = 0.22) between the two groups (Table 2). There was no difference in D′ prime (*p* = 0.43), omission (*p* = 0.53) or commission errors (*p* = 0.24) in test of attention between the two groups (Table 2).

**Table 2:** Mean differences in neurocognitive outcomes between children taking protease inhibitor and non-protease inhibitor-based antiretroviral therapy

Outcome	Antiretroviral therapy regimen		Mean difference (95% CI)	Hommel’s method corrected
	Non-PI based (*n =* 42)	PI based (*n =* 34)		*P* Value
KABC - II, mean ± SE				
Sequential processing	−0.05 (0.20)	0.85 (0.23)	− 0.90 (− 1.58, − 0.23)	0.05
Simultaneous processing	− 0.34 (0.13)	0.08 (0.15)	− 0.42 (− 0.86, 0.02)	0.19
Learning	−1.06 (0.22)	− 0.78 (0.26)	− 0.28 (− 1.02, 0.47)	0.46
Planning	− 0.31 (0.16)	0.15 (0.19)	− 0.46 (− 1.02, 0.09)	0.22
Mental processing index	− 0.95 (0.22)	− 0.41 (0.25)	− 0.55 (− 1.29, 0.19)	0.29
TOVA, mean ± SE				
Omission errors	0.30 (0.19)	0.10 (0.22)	0.20 (− 0.44, 0.84)	0.53
Commission errors	0.64 (0.20)	0.05 (0.23)	0.59 (−0.07, 1.26)	0.24
Response time total	0.27 (0.19)	−0.39 (0.21)	0.66 (0.04, 1.29)	0.15
Response time variability	0.80 (0.23)	−0.11 (0.26)	0.90 (0.13, 1.67)	0.10
D′ prime	−0.64 (0.22)	− 0.18 (0.25)	−0.45 (−1.18, 0.27)	0.43

*Abbreviations*: *PI* Protease Inhibitor, *SE* Standard error

Age-adjusted z-scores were computed using community control children from another study as the reference population and all analyses were adjusted for WHO stage at ART initiation, co-trimoxazole prophylaxis, child born before term, socioeconomic status, MUAC, and age at start of ART.

After adjusting for multiple testing using the Hommel’s method, none of the mean difference was statistically significant.

Participants in the PI group performed better than their non-PI based counterparts on motor control score (*p* = 0.016) and body coordination (*p =* 0.01), and total BOT score (*p* = 0.03). However there was no difference in manual coordination (*p* = 0.35), or strength and agility (*p* = 0.75) between the groups (Table 3).

**Table 3:** Mean differences in raw scores between children taking protease inhibitor and non-protease inhibitor-based antiretroviral therapy

Outcome	Antiretroviral therapy regimen		Mean difference (95% CI)	Hommel’s method corrected *P* value
	Non-PI based (*n* = 42)	PI based (*n* = 34)		
BOT, mean ± SE				
Total BOT score	40.51 (1.24)	46.07 (1.40)	−5.56 (−9.66, − 1.45)	0.03
Motor control score	15.54 (0.61)	18.60 (0.70)	−3.07 (−5.11, − 1.03)	0.016
Manual coordination	11.57 (0.47)	12.65 (0.54)	−1.07 (−2.64, 0.49)	0.35
Body coordination	8.00 (0.23)	9.27 (0.27)	−1.27 (−2.05, − 0.49)	0.01
Strength and agility	5.41 (0.26)	5.55 (0.30)	−0.14 (−1.02, 0.74)	0.75

*Abbreviations*: *PI* Protease Inhibitor, *SE* Standard error.

All analyses were adjusted for WHO stage at ART initiation, cotrimoxazole prophylaxis, child born before term, socioeconomic status, child’s age, and MUAC. *P* values are adjusted for multiple testing using the Hommel’s method

## Discussion

The aim of this study was to compare the neurocognitive function of children living with HIV receiving PI-based ART to those receiving non-PI-based ART. Overall, there was no difference in neurocognitive scores of children in both groups on KABC-II and TOVA. Children receiving PI-based ART performed better than their counterparts in motor coordination and body coordination and had better BOT II total scores.

The KABC II and TOVA results are similar to findings by Bangirana et al despite the longer ART exposure to participants in this study [16]. However, while Bangirana et al found no difference between the two groups in the BOT score for motor assessment, this study found that the children in PI based art arm of performed better than their counterparts. This could be because we adjusted our analysis for age at initiation of ART. More children in PI based ART group started ART at age less than 3 years compared to the non-PI based ART group.

The absence of a difference in neurocognitive scores of children in both groups on KABC-II and TOVA could be explained by the use of combined ART. The possible consequences of the low CSF concentrations of individual ART drugs may be mitigated by combining the drugs. This theory is supported by Raskino et al’s finding that combination of zidovudine and didanosine (both nucleoside reverse transcriptase inhibitors) had more neurocognitive benefit to children than didanosine monotherapy [25]. Furthermore, the study by Van den Hoff et al also suggests that despite the CSF concentration of PIs (lopinavir) being lower than those of non-PIs (Efavirez and Nevirapine), both drug concentrations were within therapeutic range [9].

The comparability of neurocognitive scores of children in both PI and non-PI groups on KABC-II and TOVA could also be explained by the fact that the children in this study had been on ART for relatively longer than otherwise younger children and had more years of school, which reduces neurocognitive impairment [11].

The findings from this pilot study imply that non-PI based ART offers no neurocognitive benefit over PI-based ART in children. However, it is possible that no difference was detected because the sample size for the study was too small to detect small differences. This is supported by the better motor coordination, body coordination and total BOT scores in the PI group compared to their non PI group, even in this small sample. A larger sample size would be able to detect small differences in neurocognitive function between the two groups. This could have significant implications on the choice of ART regimen offered to children living with HIV. The ART regimen with a neurocognitive advantage over the other gives children a better chance at a fully productive life as adults.

Despite the small sample size, this study had two important strengths. Participants had longer duration of ART on the respective treatment regimens compared to previous study by Bangirana et al, and a wide range of neurodevelopmental assessments were conducted to explore different domains of neurocognitive function. Furthermore, the tests used to assess neurocognitive function in this study have been adapted and used widely and effectively among HIV infected children in this setting, and in other countries in sub-Saharan Africa [8, 13, 20, 26].

The limitations of the study include: we did not study other factors that could affect neurocognitive function for example micronutrient and haemoglobin levels. However, from preliminary data, the prevalence of anaemia (9- < 11.5 g/dL) among children living with HIV aged 6 months to 12 years screened to participate in the ongoing “Optimizing iron status while minimizing morbidity in HIV-infected Ugandan children study (ClinicalTrials.gov Identifier: NCT03596996)” at the JCRC is relatively low at 18% based on their screening data. Furthermore, adherence to ART was done by self-report and there was no assessment of blood or CSF drug levels, so correlation with neurocognitive function could not be assessed. However, a previous study which measured CSF drug levels found no association between CSF drug levels of children and neurocognitive function [9]. CNS inflammation could result from other infections, which were not assessed in this study. We assessed the neurocognitive function at a single time point at least 1 year after ART initiation, so we are unable to comment on participants’ current scores in relation to baseline scores. We are unable to assess if baseline neurocognitive deficits persist or improve with time.

## Conclusions

There was no difference in neurocognitive test scores between children living with HIV on PI based and non-PI based ART in this pilot study. It is possible that the small sample size was contributory. The bigger prospective study would require multiple sites to achieve a large sample size.

## Supplementary Information

**Additional file 1.** Questionnaire

## Data Availability

The datasets used and/or analysed during the current study are available from the corresponding author on reasonable request.
