# Supplementary material for: Neurocognitive function among HIV-infected children on protease inhibitor -based versus non-protease inhibitor based antiretroviral therapy in Uganda: a pilot study
Source: BMC Pediatr. 2021 Apr 26;21:198. doi: 10.1186/s12887-021-02676-2 (PMC8073947; doi:10.1186/s12887-021-02676-2)
Supplement: Supplementary file 1 — Additional file 1. Questionnaire [file 12887_2021_2676_MOESM1_ESM.pdf]

Neurocognitive Function Among HIV Infected Children On Protease Inhibitor Based Versus Non Protease Inhibitor Based Antiretroviral Therapy

Questionnaire Version 1.0

October 2018

Study ID |__|__|__|__|

Complete this medical history and physical examination form at the enrolment of study participants**.**

PARTICULARS

1. Date of enrolment DD/MM/YR: ___/___/_____	2. Child's Initials:_____	3. Sex: Male [ ]<br>Female [ ]
4. Child's date of birth DD/MM/YR:___/___/_____	5. Child's Age (Completed years):	6. District of Residence:
7. Child's years of school (Completed years): ______________years	8. Caregiver's Relationship with child: ____________________	
9. Caregiver's Education level: None [ ]<br>Primary [ ]<br>Secondary [ ]<br>Tertiary [ ]<br>Don't know [ ]		

PRESENT HEALTH HISTORY

10. Is the patient currently ill with any of the following symptoms: Fever, vomiting, severe diarrhoea, active convulsions, difficulty breathing, severe cough?

N [ ] Y [ ]

If Y, send to Medical staff of JCRC for evaluation. **If N, continue with enrolment**

HIV TREATMENT HISTORY

11. Child, combination: PI-based [ ] Non-PI based [ ]

12. Adherence: How many doses have you missed in the last 3 days? ___________doses

13. ART initiation date DD/MM/YR: ___/___/_____

14. WHO clinical staging at ART initiation: I [ ] II [ ] III [ ] IV [ ]

15. Is the child on O.I prophylaxis? **N** [ ] **Y** [ ]

16. If Y, list medications:

Study ID |__|__|__|__|

PAST MEDICAL HISTORY

Has the child:

17. Any other medical condition apart from HIV? N [ ] Y [ ]
18. If yes list condition: ____
19. Been hospitalized for malnutrition? N [ ] Y [ ]
20. Been hospitalized with coma? N [ ] Y [ ]
21. Been hospitalized meningitis? N [ ] Y [ ]
22. Been hospitalized with head injury? N [ ] Y [ ]

If Yes for any of the above conditions, exclude from the study.

23. Been hospitalised for reasons other than those listed above? N [ ] Y [ ]
24. If yes, reason for the hospitalization: ____

EARLY HEALTH HISTORY

25. Was child born before term? N [ ] Y [ ]

If yes;

26. How many months early? ___________________ (completed) months
27. Birth weight in kilograms? _____________________Kg
28. For how long was the child received breast fed? __________________(completed) months

PHYSICAL EXAMINATION

29. Temp __ __. __ °C 30. Weight. __ __ . __ kg 31. Height. __ __ __ cm.
32. MUAC _______.___cm
33. Is the child acutely ill by screening exam? N [ ] Y [ ]

If yes:

Stop enrolment and refer to staff of JCRC for evaluation and treatment

GENERAL ASSESSMENT OF THE CHILD

34. General exam normal? N [ ] Y [ ]
35. If no, describe:____
36. Respiratory system normal? N [ ] Y [ ]
37. If no, describe:____
38. Cardiovascular system normal? N [ ] Y [ ]
39. If no, describe:____

Study ID |__|__|__|__|

40. GIT system normal? N [ ] Y [ ]

41. If no, describe:_______________________________________
42. CNS examination normal? N [ ] Y [ ]

43. If no, describe:_______________________________________
44. Other findings N [ ] Y [ ]
45. *If yes,* describe:_______________________________________

NEURODEVELOPMENTAL SCREEN (TQQ)

46. Compared to other children, did your child delay in sitting, standing or walking?
N [ ] Y [ ]
47. Compared to other children, does your child have difficulty seeing at night or during the day? N [ ] Y [ ]
48. Does your child seem to have difficulties hearing?
N [ ] Y [ ]
49. Does your child understand instructions?
N [ ] Y [ ]
50. Does your child have any difficultly walking, moving his/her hands, weakness or stiffness in the hands and legs?
N [ ] Y [ ]
51. Has your child ever had seizures or go into coma?
N [ ] Y [ ]
52. Did your child learn how to do things at the same time as his/her age mates?
N [ ] Y [ ]
53. Does your child speak?
N [ ] Y [ ]

If Yes:

54. Does he/she speak clearly?
N [ ] Y [ ]
55. Does he/she speak some things you can't understand at times?
N [ ] Y [ ]
56. When compared to other children of his/her age, is his/her intelligence poorer, is he/she retarded or does he/she take long to understand?
N [ ] Y [ ]

***Done by*___________________________** ***Date*___________________________**

Neurocognitive Function Among HIV Infected Children On Protease Inhibitor Based Versus Non Protease Inhibitor Based Antiretroviral Therapy					
Social Economic Status Form (1 Page)					
Study ID: |___|___|___|___|___|	*Date (DD/MM/YYYY):* |___|___|/|___|___|/|___|___|___|___|				
EDUCATION					
1. Is the child currently in school?	☐ *YES*	☐ *NO*			
2. If YES, child is in school, what level?	☐ *0-none*	☐ *1-preschool*	☐ *2-nursery*	☐ *3-P.1-P4*	☐ *4-P.5-P7*
3. If NO, was child ever in school?	☐ *YES*	☐ *NO*			
4. If YES, child WAS in school, what level?	☐ *0-none*	☐ *1-preschool*	☐ *2-nursery*	☐ *3-P.1-P.4*	☐ *4-P.5-P7*
5. Who is the child's primary caregiver?	☐ *Father*	☐ *Mother*	☐ *Other, specify*______________________ __________		
6. Is child's mother able to read and write?	☐ *YES*	☐ *NO*			
7. What was the highest level of education for the child's primary care giver?	☐ *0-none*	☐ *1-primary*	☐ *2-secondary*	☐ *3-tertiary*	
8. Is child's father able to read and write?	☐ *YES*	☐ *NO*	☐ *Don't know*		
9. What was highest level of education for child's father?	☐ *0-none*	☐ *1-primary*	☐ *2-secondary*	☐ *3-tertiary*	☐ *4-don't know*

Socio-Economic Status													
1. How many siblings does this child have?													
☐ *0*	☐ *1*	☐ *2*	☐ *3*	☐ *4*	☐ *5*	☐ *6*	☐ *7*	☐ *8*	☐ *9*	☐ *10*	☐ *11*	☐ *12*	☐ *>12*
2. How many people live under the same roof as this child?													

Study ID |__|__|__|__|

Neurocognitive Function Among HIV Infected Children On Protease Inhibitor Based Versus Non Protease Inhibitor Based Antiretroviral Therapy													
☐ 0	☐ 1	☐ 2	☐ 3	☐ 4	☐ 5	☐ 6	☐ 7	☐ 8	☐ 9	☐ 10	☐ 11	☐ 12	☐ >12

3. What type of roof do you have?	☐ 1-Thatch	☐ 2-Iron sheets	☐ 3-Tiles	☐ 4-Don't know
4. What kind of water supply do you have?	☐ 1-Stream/pond/lake	☐ 2-Well	☐ 3-Borehole	☐ 4-Tap near or inside home
5. What kind of cooking fuel do you use?	☐ 1-Firewood	☐ 2-Charcoal	☐ 3-Paraffin	☐ 4-Gas/Electricity
6. Does the family eat meat at least once a week?	☐ YES	☐ NO		
7. Does the family have food all year round?	☐ YES	☐ NO		

8. Which of the following items are owned by you or found in your home? Add scores for YES responses for total

Item			*Score*	*Item*			*Score*
Electricity	☐ YES	☐ NO	3	*Bicycle*	☐ YES	☐ NO	1
Shoes for subject	☐ YES	☐ NO	1	*Motorcycle*	☐ YES	☐ NO	2
Radio	☐ YES	☐ NO	1	*Motor vehicle*	☐ YES	☐ NO	3
Television	☐ YES	☐ NO	2	*Animals (>2: cows, goats, pigs)*	☐ YES	☐ NO	2
				Poultry >10	☐ YES	☐ NO	2
TOTAL (add scores from both columns of all YES responses)				|____|____|			

MIGRATION	
In which district was the subject born?	*District:*

Study ID |__|__|__|__|

Neurocognitive Function Among HIV Infected Children On Protease Inhibitor Based Versus Non Protease Inhibitor Based Antiretroviral Therapy			
In which district has the child lived for most of life?	***District:***		
In which district did the subject usually live for past 12 months?	***District:***		
FORM COMPLETED BY:	|___|___|___|	***SIGNATURE***	
DATE (DD/MM/YYYY)	|____|____| / |____|____| / |____|____|____|____|		
